# Formulation Optimization of Sinomenine-Loaded Nanostructured Lipid Carriers Based on Molecular Dynamics Simulations and Evaluation of Pharmacological Efficacy in Rheumatoid Arthritis

**DOI:** 10.3390/ijms262311449

**Published:** 2025-11-26

**Authors:** Xinmeng Lin, Xuehui Ding, Yunlu Zou, Mingyang Sheng, Jingying Li, Yinghao Xiao, Jiahui Xu, Jixin Li, Lin Wang, Wei Xu

**Affiliations:** School of Pharmacy, Changchun University of Chinese Medicine, Changchun 130117, China; 18043639284@163.com (X.L.); 15837573625@163.com (X.D.); 17390991885@163.com (Y.Z.); 16643007923@163.com (M.S.); 18043910903@163.com (J.L.); 13517376162@163.com (Y.X.); 15590142627@163.com (J.X.); 18343412752@163.com (J.L.)

**Keywords:** Sinomenine, nanostructured lipid carrier, molecular dynamics simulations, pharmacodynamics, single-pass intestinal perfusion

## Abstract

Sinomenine (SIN), as a potential therapeutic agent for rheumatoid arthritis (RA), exhibits advantages such as non-addictiveness. However, its low aqueous solubility and poor membrane permeability result in limited bioavailability, which compromises its therapeutic efficacy in conventional formulations. To address these limitations, this study developed nanostructured lipid carriers (NLCs) with optimized formulations and evaluated their pharmacodynamic performance. Molecular dynamics (MD) simulations were employed to screen excipients and analyze the blending system. SIN-loaded NLCs (SIN-NLCs) were prepared using high-pressure homogenization. Single-factor experiments were performed to optimize the processing conditions of SIN-NLCs. A three-factor, three-level experimental design was established using Design Expert 13 software and further refined through Box–Behnken design (BBD) response surface methodology. This approach enabled cross-validation between molecular dynamics simulations and conventional experiments. Additionally, transmission electron microscopy (TEM) was used to examine morphology, while X-ray diffraction analysis (XRD), differential scanning calorimetry (DSC), and Fourier-transform infrared spectroscopy (FT-IR) were employed to characterize the physicochemical state of SIN in NLCs. Pharmacodynamic evaluation was performed in a RA model, supplemented by single-pass intestinal perfusion study (SPIP). Initially, MD simulations were employed to evaluate drug–excipient compatibility, thereby identifying suitable formulation excipients: stearic acid and oleic acid as lipid components, and Poloxamer 188 as the surfactant. Subsequently, single-factor experiments combined with the BBD response surface methodology were employed to optimize preparation parameters, establishing the ideal process conditions: drug-to-lipid ratio of 1:42, solid-to-liquid lipid ratio of 5.58:4.42, and Poloxamer 188 concentration of 1.20%. The optimized SIN-NLCs exhibited spherical particles with uniform dispersion and no agglomeration. The average particle size was 173.90 ± 1.97 nm, with a polydispersity index (PDI) of 0.18 ± 0.01, a zeta potential of −22.65 ± 0.60 mV, and an encapsulation efficiency (EE%) of 91.27% ± 0.01. Spectroscopic analysis confirmed that SIN existed in an amorphous state and was successfully encapsulated within the lipid matrix. In vivo, SIN-NLCs significantly reduced paw swelling and arthritis scores in model rats, promoted synovial cell proliferation, and suppressed inflammatory cell infiltration. The intestinal perfusion study demonstrated that SIN-NLCs were primarily absorbed in the small intestine and markedly enhanced drug permeability. SIN-NLCs represent an effective delivery system to enhance the solubility and permeability of SIN. This study provides a novel strategy and methodology for the formulation of hydrophobic drugs, offering valuable insights for future pharmaceutical development.

## 1. Introduction

Rheumatoid arthritis (RA) is an autoimmune disease characterized by chronic synovial inflammation and progressive cartilage destruction, which can lead to joint deformities if not treated promptly [1]. Current therapeutic options, however, struggle to balance efficacy and safety: non-steroidal anti-inflammatory drugs only relieve pain and inflammation and may cause gastrointestinal ulceration and renal damage with long-term use [2]; conventional disease-modifying antirheumatic drugs such as methotrexate require 3–6 months to take effect and may induce hepatic, renal, and hematopoietic toxicity [3]; biologics offer high target specificity but are costly, may increase infection risk, and some patients develop resistance [4]; and glucocorticoids provide rapid anti-inflammatory action but lead to adverse effects such as osteoporosis and glucose dysregulation, limiting long-term application [5]. These shortcomings highlight the need to develop new drug-delivery systems.

Sinomenine (SIN) is a key active alkaloid of the morphinan-type, exhibiting multiple pharmacological activities. It exerts analgesic, anti-inflammatory, and immunosuppressive effects [6], demonstrating significant therapeutic potential for diseases such as RA [7]. However, its clinical application is hindered by poor water solubility and limited permeability. Biopharmaceutical limitations arising from poor solubility or permeability necessitate formulation strategies such as salt formation [8], nanotechnology [9], inclusion complexes [10], and modification of crystallization behavior [11]. In recent years, nanomedicines have emerged as novel drug delivery systems [12], enhancing the medical application of poorly soluble drugs through diverse formulations and technologies. Nanostructured lipid carriers (NLCs) can enhance the stability of encapsulated drugs against oxidation and degradation, improve their aqueous solubility, control the drug release rate in vivo, and ultimately increase their bioavailability [13,14,15,16]. NLCs are characterized by a disordered crystal structure, which provides high physical stability, minimal drug leakage, and a controllable release profile, rendering their performance comparable to that of polymeric nanoparticles. NLCs exhibit improved controlled drug release and enhanced membrane permeability in the gastrointestinal tract. Consequently, they represent a promising novel delivery system [17]. Considering the long-term medication requirements of rheumatoid arthritis patients and the advantages of NLCs, this study developed orally administered SIN-loaded NLCs (SIN-NLCs) to improve treatment adherence. NLCs can encapsulate degradation-prone drugs and protect them from gastrointestinal inactivation [18], while their small particle size enhances intestinal absorption, reduces gastrointestinal irritation and adverse reactions, and ultimately improves the overall safety of therapy [19].

In recent years, molecular dynamics (MD) simulations have gained increasing adoption in pharmaceutical formulation development. By mimicking laboratory conditions at the molecular level, this computational technique enables accurate characterization of microscopic dynamic behaviors within target systems and offers mechanistic interpretations of experimental observations—thereby improving the efficiency, cost-effectiveness, and predictive power of related research. Notably, MD simulations were utilized for excipient screening and investigation of solid–liquid mixing systems [20]. Shen et al. proposed using MD simulations to predict drug–excipient compatibility and estimate solid–liquid lipid ratios [21]. This provides deeper insights into molecular interactions and expands the potential for formulation screening. Based on MD simulations screening, an optimized formulation was developed. Experimental results further validated the accuracy predicted by MD simulations. This study employs a combined approach of MD simulations and conventional experiments. This approach facilitates formulation research, advances the development of pharmaceutical technologies, and provides a methodological framework for pharmaceutical research. Moreover, this study provides important potential and a practical foundation for the subsequent development of efficient and low-toxicity oral formulations for the treatment of RA.

## 2. Results

### 2.1. Excipient Screening

The magnitude of intermolecular cohesive energy (CED) was evaluated by the solubility parameters (δ), which reflects the compatibility between blend components. Table 1 summarizes the calculated CED and δ values for the drug with different excipient carriers, together with literature values for comparison.

As shown in Table 1, the results of MD simulations agreed well with literature values, indicating that this method is suitable for the quantitative calculation of solubility parameters in this system. A smaller Δδ value corresponds to better compatibility between the carrier and the drug. When the Δδ between two components is <7.0 (J/cm^3^)^1/2^, the drug and carrier are considered compatible [22]. Based on this criterion, the drug exhibited good solubility in lipid carriers, surfactants, and organic solvents, but not in water. The solubility order of SIN in solid lipid carriers was stearic acid > GMS > glycerol tristearate; in liquid lipids: oleic acid > glycerol tricaprylate > α-tocopherol; in surfactants: Poloxamer 188 > lecithin > PVA 0486; and in organic solvents: ethanol > water. From a compatibility perspective, NLCs were therefore formulated using SIN as the model drug, stearic acid and oleic acid as lipid carriers, Poloxamer 188 as the surfactant, and ethanol as the organic solvent.

### 2.2. Simulation of Lipid Carrier Blending Systems

**Figure 1:**
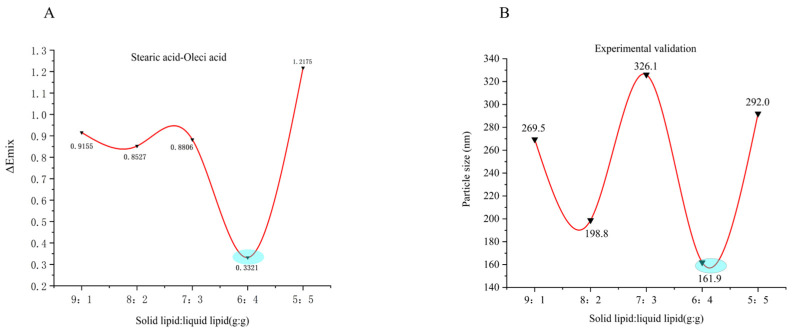
Predictions and traditional test results. (**A**) ΔE_mix_ values of the blend system at different mixing ratios; (**B**) Particle size analysis of the blend system at different mixing ratios.

As shown in Figure 1A, lower mixing energy (ΔE_mix_) corresponds to greater stability of the blend system [23]. Accordingly, the stearic acid-oleic acid blend system was found to be compatible. When an appropriate solid-to-liquid lipid ratio is maintained, phase separation is less likely to occur during preparation and storage, thereby improving drug encapsulation efficiency and overall formulation stability. Within this blend system, the ΔE_mix_ value reached a minimum at an oleic acid content of 40%, suggesting optimal stability under these conditions. Consistently, the particle size distribution results (Figure 1B) showed a narrower distribution at 40% oleic acid content, in agreement with the MD simulations findings.

### 2.3. Formulation and Process Optimization of SIN-NLCs

#### 2.3.1. Single-Factor Optimization Formulation

**Figure 2:**
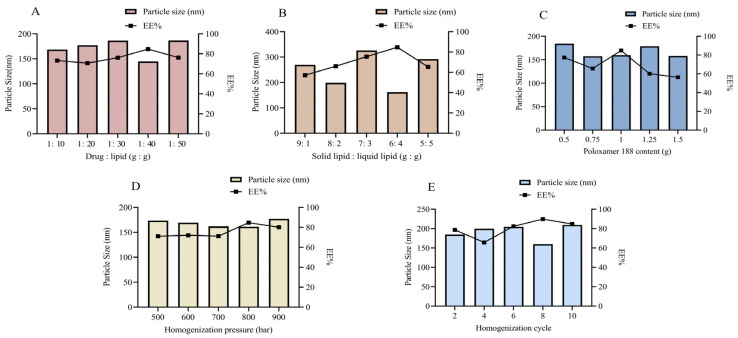
Formulation screening of SIN-NLCs. (**A**) Effect of different drug-lipid ratios (g: g) on particle size and EE%; (**B**) Effect of solid-to-liquid lipid ratio (g: g) on particle size and EE%; (**C**) Effect of Poloxamer 188 content (%) on particle size and EE%; (**D**) Effect of homogenization pressure (bar) on particle size and EE%; (**E**) Effect of homogenization cycles on particle size and EE%. Data are presented as the mean ± standard deviation (n = 3).

Since formulation and process parameters critically influence the drug loading capacity and release profile of NLCs, screening was performed to optimize the NLC system. Various formulations were screened based on characterization parameters. The effect of different drug-to-lipid mass ratios on drug content was evaluated (Figure 2A). Results showed that the encapsulation efficiency (EE%) decreased when the drug-to-lipid mass ratio exceeded 1:40. The primary reason is that an excessively low drug loading reduces the dispersion of the drug within the lipid matrix, making it difficult for the lipid phase to achieve adequate encapsulation. Moreover, an excessive amount of lipid matrix can lead to a loosened carrier structure, thereby increasing the risk of drug leakage [24]. We propose that at higher drug doses, the drug content exceeds the capacity of the lipid blend, leading to reduced EE%. The particle size was also smallest at a drug-to-lipid ratio of 1:40, making this ratio optimal. To determine the best ratio of stearic acid to oleic acid, the solid-to-liquid lipid ratio was evaluated across multiple blend systems (Figure 2B). No lipid separation was observed at a solid-to-liquid lipid ratio of 6:4. NLCs prepared at this ratio exhibited the smallest particle size and highest EE%, which was further validated by the previously mentioned MD simulations findings. Emulsifiers play an essential role in reducing particle size and enhancing nanoparticle stability by covering the surface of hydrophobic particles, lowering surface tension between the particles and the aqueous medium, and preventing aggregation. The surfactant content also significantly influenced particle size and EE% (Figure 2C). The smallest particle size and highest EE% were achieved with 1.00% Poloxamer 188 content. Excessively high content of Poloxamer 188 can lead to drug precipitation and micelle aggregation, compromising the stability of the blend system. Therefore, 1.00% Poloxamer 188 was selected as the optimal formulation.

During SIN-NLCs preparation via high-pressure homogenization, both homogenizer pressure and cycle number influence particle size and distribution. As shown in Figure 2D, the smallest SIN-NLC particle size was achieved at an average pressure of 800 bar. As depicted in Figure 2E, the smallest particle size with the most uniform distribution was obtained after 8 cycles. Thus, a homogenization pressure of 800 bar with 8 cycles represents the optimal homogenization conditions for the formulation.

#### 2.3.2. Box–Behnken Design (BBD) Experiment for Further Optimization of the Formulation

Based on the results of the single-factor experiments, the drug-lipid ratio, solid-to-liquid lipid ratio, and Poloxamer 188 mass fraction were selected as primary factors for a BBD experiment. Encapsulation efficiency and particle size of SIN-NLCs were used as response variables for optimizing the preparation process. Design factors are shown in Table 2.

**Figure 3:**
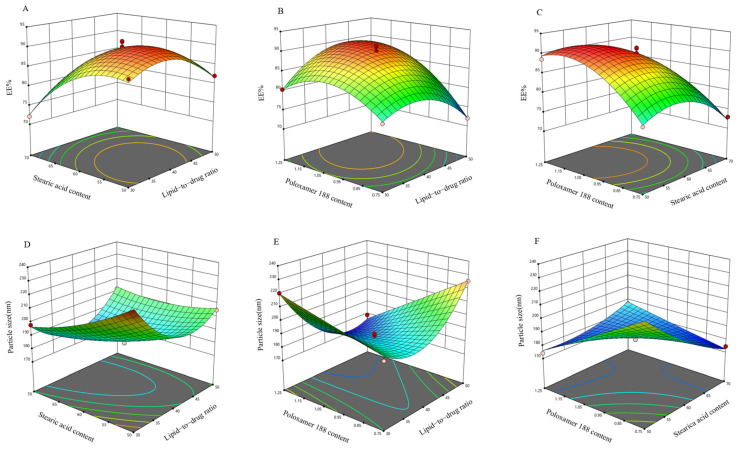
Response surface plots illustrating the interactions between key formulation factors. (**A**) Interaction between drug-to-lipid ratio and solid-to-liquid lipid ratio on EE%; (**B**) Interaction between drug-to-lipid ratio and Poloxamer 188 content on EE%; (**C**) Interaction between solid-to-liquid lipid ratio and Poloxamer 188 content on EE%; (**D**) Interaction between drug-to-lipid ratio and solid-to-liquid lipid ratio on particle size; (**E**) Interaction between drug-to-lipid ratio and Poloxamer 188 content on particle size; (**F**) Interaction between solid-to-liquid lipid ratio and Poloxamer 188 content on particle size.

Using Design-Expert 13.0 software, response surface plots (Figure 3) were generated based on the regression equation, following analysis of variance results for the regression model. Steeper response surface curves indicate more pronounced factor effects. The optimized formulation was determined to have a drug-to-lipid ratio of 1:42, a solid-to-liquid lipid ratio of 5.58:4.42, a Poloxamer 188 content of 1.20%, an encapsulation efficiency of 91.27%, and a particle size of 173.90 nm. Three parallel batches of SIN-NLCs were prepared using the optimized process. The measured average encapsulation efficiency was 90.78%, and the particle size was 174.10 nm, closely matching the predicted encapsulation efficiency of 91.27% and particle size of 173.90 nm, indicating that this process is stable and feasible.

### 2.4. Characterization of SIN-NLCs

#### 2.4.1. Appearance, Zeta Potential, and Particle Size Distribution

**Figure 4:**
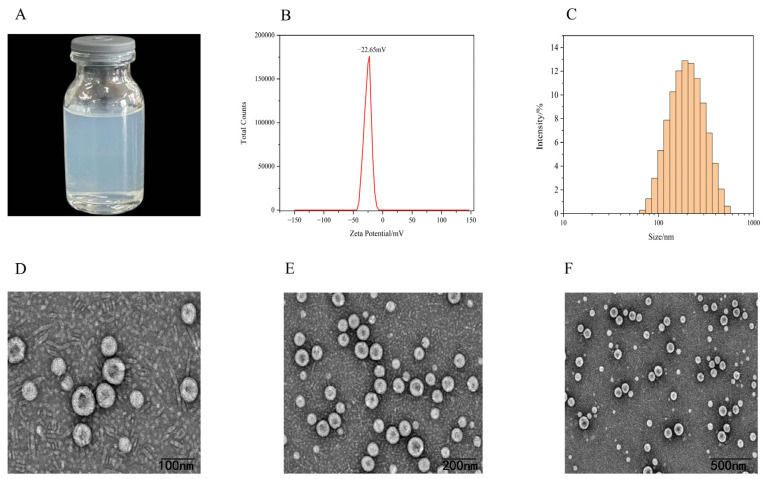
Characterization of SIN-NLCs. (**A**) Visual appearance; (**B**) Zeta potential profile; (**C**) Particle size distribution curve; (**D**–**F**) TEM images of SIN-NLCs observed at nanoparticle sizes of approximately 100 nm, 200 nm, and 500 nm, respectively.

The resulting SIN-NLCs appeared as a transparent light blue liquid with no stratification or precipitation, exhibiting excellent flowability (Figure 4A). The negative zeta potential of −22.65 ± 0.60 mV (Figure 4B) indicated the physical stability of the nanoscale system. The average particle size of SIN-NLCs measured by dynamic light scattering (DLS) was 174.10 ± 1.97 nm (Figure 4C), with a polydispersity index (PDI) of 0.18 ± 0.01. The PDI is used to determine the uniformity of particle size distribution. A PDI value below 0.5 indicates a controlled-release particle size distribution in NLCs. This narrow and uniform distribution increases the drug’s surface area and wettability, leading to enhanced solubility in the gastrointestinal tract and promoting drug absorption according to the Noyes-Whitney equation [18].

#### 2.4.2. Transmission Electron Microscopy (TEM)

As shown in Figure 4D–F, TEM images reveal that the particles exhibit a regular spherical morphology with good dispersion and no agglomeration.

### 2.5. Spectral Analysis of SIN-NLCs

#### 2.5.1. X-Ray Diffraction Analysis (XRD)

**Figure 5:**
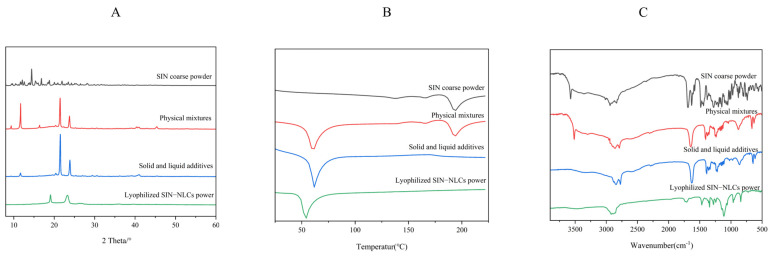
Spectral analysis of SIN-NLCs. (**A**) XRD patterns of SIN coarse powder, physical mixture, solid–liquid excipient, and SIN-NLCs; (**B**) DSC curves of SIN coarse powder, physical mixture, solid–liquid excipient, and SIN-NLCs; (**C**) FT-IR spectra of SIN coarse powder, physical mixture, solid–liquid excipient, and SIN-NLCs.

The XRD patterns of the pure components and physical mixtures show distinct peaks, indicating different characteristics and crystal structures [25] (Figure 5A). The SIN diffraction peaks at 2θ = 16.8°, 18.8°, 19.9°, 21.9°, and 23.4° are prominent, with additional weaker peaks at other positions. Compared to SIN, the SIN-NLCs diffraction patterns show identical peaks at 19.1° and 23.2°. In contrast, the physical and solid–liquid mixtures exhibit an additional diffraction peak at 11.6° relative to SIN-NLCs. These results suggest that SIN is not crystalline within the lipid matrix, but rather exists as non-crystalline forms or individual molecules dissolved in the matrix.

#### 2.5.2. Differential Scanning Calorimetry (DSC)

DSC is a fundamental technique for studying phase transition temperatures and energy changes in drug mixtures [26]. Figure 5B shows the DSC analysis of SIN. It reveals a melting peak for SIN at 194.7 °C. The physical mixtures show a maximum peak at 60.2 °C, whereas SIN-NLCs exhibit a melting peak at 54.1 °C. The DSC curve of SIN-NLCs did not show an endothermic peak for SIN. The peak intensity was lower and the peak width broader compared to the physical mixture. The shift in the melting point may result from interactions between solid and liquid lipids during preparation, forming a more disordered crystalline structure. This further confirms the encapsulation of SIN within NLCs. Furthermore, the SIN peak at 194.7 °C disappeared in the SIN-NLCs spectrum, indicating that SIN was captured by NLCs and existed in an amorphous state.

#### 2.5.3. Fourier-Transform Infrared Spectroscopy (FT-IR)

FT-IR spectroscopy was employed to investigate molecular interactions between SIN and the lipid matrix. As shown in Figure 5C, characteristic absorption bands of SIN include the -OH stretching vibration at 3571.6 cm^−1^, C-H stretching vibrations at 2939.9 cm^−1^, C=O and C=C stretching vibrations at 1689.3 cm^−1^, skeletal vibrations of aromatic rings and bending vibrations of methyl and methylene groups at 1485.0 cm^−1^, and additional bands at 1280.8, 1202.8, and 1147.5 cm^−1^. In the FT-IR spectra of the physical mixture and SIN-NLCs, peaks at 2920.5, 2851.0, 1703.5, 1462.4, and 940.4 cm^−1^ correspond to the lipid matrix. Notably, in the SIN-NLCs spectrum, the characteristic -OH stretching vibration peak of SIN near 3571.6 cm^−1^ disappears, confirming successful encapsulation of SIN in the lipid carrier.

### 2.6. In Vivo Pharmacodynamic Studies

#### 2.6.1. Paw Swelling Rate in Rats

**Figure 6:**
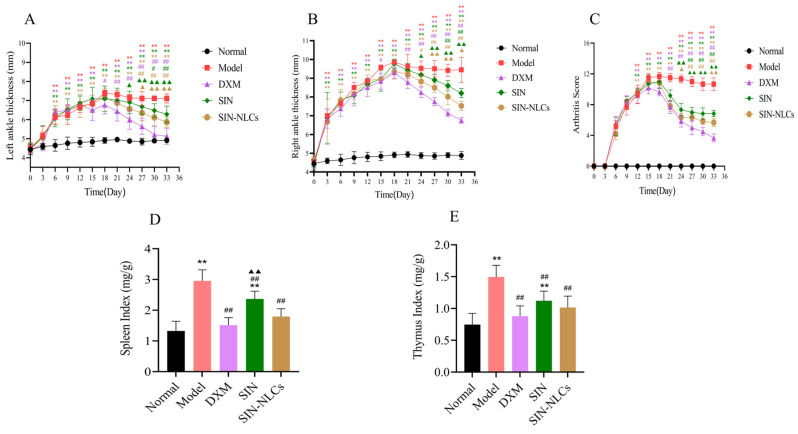
Analysis of paw swelling, arthritis scores, and immune organ indices in rats. (**A**) Left paw swelling; (**B**) Right paw swelling; (**C**) Arthritis score chart; (**D**) Spleen index; (**E**) Thymus index. (n = 6, ** *p* < 0.01, compared with the Normal group; ^#^ *p* < 0.05, ^##^ *p* < 0.01, DXM group, SIN group and SIN-NLCs group respectively compared with the Model group; ^▲^ *p* < 0.05, ^▲▲^ *p* < 0.01 SIN and SIN-NLCs groups compared with DXM group respectively).

An RA rat model was established, and the effects of SIN-NLCs were evaluated by measuring swelling rate and arthritis score. From day 7 after modeling, rats in both the model and treatment groups showed significant joint swelling compared with the normal group. The swelling rate peaked on day 18 (*p* < 0.01), confirming successful establishment of the RA model. After treatment, joint swelling was significantly reduced in the SIN-NLCs, SIN, and Dexamethasone (DXM, positive control group) compared with the model group (*p* < 0.05, *p* < 0.01), as shown in Figure 6A,B.

#### 2.6.2. Arthritis Scoring in Rats

On day 18 post-primary immunization, rats exhibited peak arthritis scores, characterized by severe joint swelling and notable lameness. The scores in the model group were significantly higher than those in the normal group (*p* < 0.01). After 15 days of treatment, arthritis scores significantly decreased in all groups (SIN-NLCs, SIN, and DXM), with the SIN-NLCs group showing superior efficacy compared to the SIN group (*p* < 0.01; Figure 6C).

#### 2.6.3. Immune Organ Index in Rats

Compared to the normal group, the model group showed significant increases in thymus and spleen indices (*p* < 0.01). Treatment with SIN-NLCs, SIN, or DXM markedly reduced these elevated indices relative to the model group, with the SIN-NLCs group exhibiting markedly lower indices than the SIN group (*p* < 0.01; Figure 6D,E).

#### 2.6.4. Inflammatory Mediators in Rats

**Figure 7:**
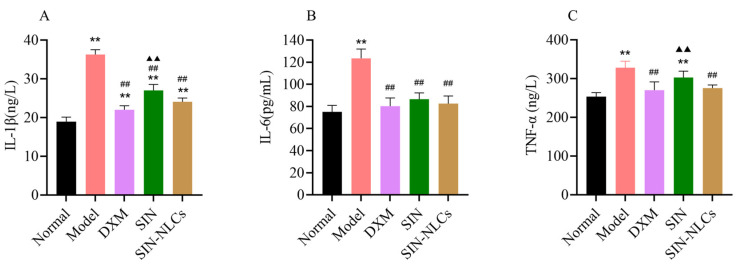
Inflammatory Cytokine Profile in Rats. (**A**–**C**) Levels of proinflammatory cytokines IL-1β, IL-6, and TNF-α in rat serum were detected using enzyme-linked immunosorbent assay (ELISA) kits. (** *p* < 0.01, compared with the Normal group; ^##^ *p* < 0.01, DXM group, SIN group and SIN-NLCs group respectively compared with the Model group; ^▲▲^ *p* < 0.01 SIN and SIN-NLCs groups compared with DXM group respectively).

Compared with the SIN group, the SIN-NLCs group exhibited lower concentrations of interleukin 1β (IL-1β), Serum levels of interleukin-6 (IL-6) and tumor necrosis factor-α (TNF-α), indicating that this group significantly suppressed the secretion of proinflammatory cytokines and alleviated the severity of RA, as shown in Figure 7A–C.

#### 2.6.5. Histopathological Examination (HE)

**Figure 8:**
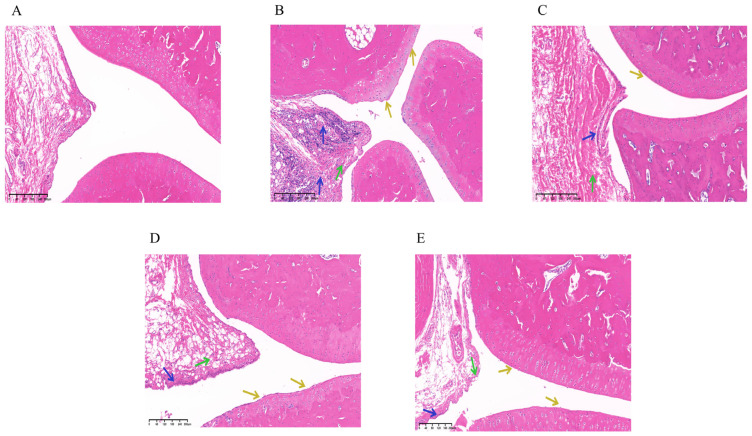
Arthritis tissue sections from each rat group. (**A**) Normal group; (**B**) Model group; (**C**) Dexamethasone group; (**D**) SIN group; (**E**) SIN-NLCs group. Green arrows: Synovial cell nuclei, Yellow arrows: Chondrocytes, Blue arrows: Inflammatory cells.

HE staining results demonstrated that the normal group exhibited intact tissue architecture, with synovial cells displaying well-defined, spindle- or oval-shaped nuclei, and no signs of necrosis or hyperplasia. Chondrocytes were situated within cartilage pits, and the cartilage layer maintained normal thickness with a smooth surface. No significant inflammatory cell infiltration was observed. In contrast, the model group showed moderately abnormal tissue morphology. Synovial cell nuclei remained visible, with spindle- or oval-shaped morphology, but marked proliferation was evident (indicated by green arrows). Chondrocytes remained within cartilage pits, and cartilage thickness was preserved; however, the cartilage surface appeared relatively uneven (yellow arrows). Notably, substantial inflammatory cell infiltration was present (blue arrows). Compared to the model group, both the SIN-NLCs and DXM groups significantly alleviated inflammation, reduced synovial cell proliferation, and resulted in a smoother cartilage surface. These findings suggest that SIN-NLCs effectively mitigate pathological alterations in joint tissues and attenuate the severity of RA. As shown in Figure 8. At the end of the experimental study, none of the treatment groups exhibited a complete reversal of arthritis-induced pathological alterations across all measured parameters.

### 2.7. Single-Pass Intestinal Perfusion Study (SPIP)

**Figure 9:**
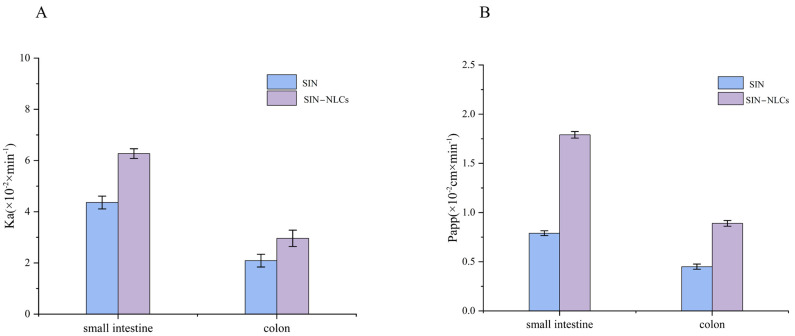
Comparison of in situ absorption of SIN solution and SIN-NLCs solution in rat intestines. (**A**) Absorption rate Ka; (**B**) Effective permeability coefficient Papp. Results are expressed as the mean ± standard deviation (n = 3).

Studies indicated that SIN remained stable in infusion solutions prior to administration. Investigating the intestinal absorption characteristics and mechanisms of drugs is essential for the design and evaluation of oral formulations. The SPIP method was employed to compare the absorption properties of the SIN solution and the SIN-NLC solution in the small intestine and colon. The absorption rate constant (Ka) and effective permeability coefficient (Papp) values derived from SPIP are shown in Figure 9. Results demonstrated that SIN exhibited significantly higher Ka and Papp values in the small intestine than in the colon. Similarly, the Ka of SIN-NLCs in the small intestine was significantly higher than that in the colon. Specifically, the Ka value of SIN-NLCs increased from 4.36 × 10^−2^ to 6.27 × 10^−2^ min^−1^, representing a 1.44-fold increase compared to that of the SIN solution. Correspondingly, the Papp value of SIN-NLCs in the small intestine was approximately 2.26 times greater than that of the SIN solution. These findings suggested that SIN-NLCs are primarily absorbed in the small intestine.

## 3. Discussion

Through extensive database and literature searches involving hundreds of excipients, we preliminarily screened matrices suitable for preparing NLCs. Subsequently, MD simulations were conducted on 11 candidate surfactants to calculate their δ and CED, which facilitated the selection of optimal surfactants for SIN-NLCs formulation. The lipid matrix comprised stearic acid and oleic acid, with Poloxamer 188 as the surfactant and ethanol serving as the solvent. Using the Flory-Huggins theory, the mixing energy of the system was calculated at various lipid ratios. Results indicated the highest stability at a solid-to-liquid lipid ratio of 6:4. Particle size analysis from conventional experiments confirmed that this ratio produced higher encapsulation efficiency and a smaller, more uniform particle size distribution, validating the MD simulations’ predictions. This integrated approach of theoretical modeling with experimental validation notably shortened the formulation development timeline while highlighting the value of MD techniques in pharmaceutical research. XRD analysis revealed the absence of characteristic crystalline peaks of SIN within the SIN-NLCs powder, indicating that SIN existed in an amorphous state inside the lipid matrix. DSC results showed a melting point lower than that of the pure lipids, attributable to changes in crystallinity and melting behavior caused by active molecules or liquid lipids within the lipid matrix [27]. FT-IR spectroscopy confirmed successful encapsulation, as evidenced by the disappearance of the characteristic SIN -OH stretching vibration near 3571.6 cm^−1^ in the SIN-NLCs spectrum, indicating effective integration of SIN within the lipid carrier. The optimized SIN-NLCs were subsequently characterized for physicochemical properties, including particle size and morphology, demonstrating a stable and uniformly dispersed nanocarrier system.

In this study, the SIN-NLCs group showed a significant reduction in the arthritis index and alleviated toe redness and swelling, suggesting effective suppression of inflammatory symptoms in RA rats. Histopathological analysis revealed that, in the SIN-NLCs group, cell arrangement was orderly, synovial cell proliferation was decreased, and inflammatory cell infiltration was minimal. These changes were markedly different from those observed in the model group, indicating notable therapeutic benefits. The pathogenesis of RA is complex and involves increased infiltration of T and B lymphocytes in joint effusions and synovial tissues, which promote the secretion of various inflammatory cytokines. Key cytokines, including IL-1β, IL-6, and TNF-α, significantly influence disease onset and progression. Notably, IL-1β, chiefly produced by macrophages, plays a pivotal role by activating monocyte-macrophages, inducing adhesion molecules, chemokines, and other mediators that exacerbate joint inflammation [28]. The present study demonstrated that the formulation reduced serum levels of IL-1β, IL-6, and TNF-α, suggesting that its anti-RA effects may involve downregulating inflammatory cytokine production and alleviating paw swelling, thus exerting anti-inflammatory and immunomodulatory actions [29].

Statistical analysis showed no significant difference between the SIN-NLCs and DXM groups in primary pharmacodynamic endpoints (*p* > 0.05), indicating comparable efficacy. In terms of safety and physiological status, significant differences were observed between the two groups. Some rats in the DXM group even exhibited mild diarrhea. In contrast, rats in the SIN-NLCs group showed no abnormal physiological responses and exhibited no significant differences compared to the Normal group. These findings indicate that, unlike the DXM group, SIN-NLCs do not adversely affect growth or overall health. However, the DXM group, as a positive control, has notable limitations: its poor water solubility, low bioavailability, and lack of targeting require repeated high doses, which increase side effects. Dexamethasone is associated with adverse events such as immunosuppression, adrenal insufficiency, gastrointestinal ulcers, osteoporosis, and atrophy of immune organs, potentially confounding experimental outcomes or necessitating animal sacrifice [30]. The adverse effects observed in the DXM group may be attributed to the well-documented side effects of glucocorticoids, including suppression of the hypothalamic–pituitary–adrenal axis leading to adrenal cortical insufficiency, inhibition of gastrointestinal motility with reduced food intake, and disruption of energy metabolism through altered glucose and insulin regulation [31]. In summary, while SIN-NLCs exhibited anti-inflammatory efficacy comparable to Dex, they were associated with no detectable adverse reactions, indicating superior safety and biocompatibility in anti-inflammatory therapy.

SPIP studies demonstrate that several factors may contribute to the enhanced absorption of SIN-NLCs in rats. First, the nanoscale particle size markedly increases the surface area of the drug, thereby significantly elevating Ka and Papp values for both SIN and SIN-NLCs solutions [32]. Second, after oral administration, the lipid carrier is mainly digested by lipases to form micelles or vesicles, which readily traverse the aqueous layer to facilitate absorption in the small intestine [33]. The lipid composition of the carrier shares similarities with that of intestinal epithelial cell membranes, increasing affinity and promoting drug transport [34]. Third, the polyoxyethylene chains in Poloxamer 188 may transiently alter the lipid structure of intestinal mucosal membranes, increasing membrane fluidity and permeability, thus facilitating drug passage into the bloodstream [35]. Lastly, the physiological structure of the small intestine, characterized by folds and villi, considerably enlarges the surface area of the intestinal lumen, enhancing drug absorption and uptake by the mucosa [36]. NLCs significantly enhance drug penetration and absorption. This effect is likely due to their smaller particle size and uniform distribution, which increase contact with the gastrointestinal mucosa and facilitate drug uptake. Additionally, NLCs reduce SIN degradation within the intestine, improving its stability and leading to increased systemic circulation [37]. Components of the formulation, such as stearic acid, oleic acid, and Poloxamer 188, may also contribute to enhanced drug absorption [38].

## 4. Materials and Methods

### 4.1. Materials

Sinomenine (SIN, 98%, Chengdu Chenye Biotechnology Co., Ltd., Chengdu, China), oleic acid, Stearic acid and Poloxamer 188 (Shanghai Yuanye Biotechnology Co., Ltd., shanghai, China) conform to the standards for analytical grade purity and quality. Specific-pathogen-free (SPF)-grade Sprague Dawley (SD) male rats, weighing 200 ± 20 g, were obtained from Liaoning Changsheng Biotechnology Co., Ltd., Liaoning, China, (License No.: SCXK(Liao)2020-0001). The animal experiment protocol was approved by the Animal Ethics Committee of Changchun University of Chinese Medicine (Approval No.: 2024796), in accordance with institutional guidelines.

### 4.2. Excipient Screening

MD simulations were employed to calculate the δ of pure substances [39] and to evaluate the compatibility between drugs and excipients [40]. First, the Pharma DE expert system (https://pharmde.computpharm.org/) (accessed on 26 March 2024) was used to perform risk assessments of drugs and excipients, thereby identifying low-risk excipients. Subsequently, the Visualizer module within the MD simulations software (The calculations were performed using GROMACS 2022.5 software on a Lenovo server (16-core, 32-thread, Linux operating system) at Lenovo Group Limited in Beijing, China.) was used to construct monomer models for SIN, lipid carriers, surfactants, and solvent molecules. After constructing the molecular models, each structural unit underwent energy minimization to eliminate unfavorable initial intermolecular contacts and obtain a more reasonable molecular structure. The Discover module was used to optimize the molecular models: the steepest descent method adjusted the initial molecular structure, followed by refinement with the conjugate gradient method, and convergence was achieved using the Newton-Raphson method. Amorphous structural units were constructed using periodic boundary conditions. Each amorphous structural unit underwent 5000 optimization steps. An NVT ensemble was selected, and 16,000 MD simulation steps were performed using an Andersen thermostat. The lowest-energy framework was selected for MD annealing until the system energy plateaued. Subsequently, an NVT ensemble was employed with an Andersen thermostat at 298 K. The time step was set to 1 fs, with a total simulation length of 200,000 steps. The initial 100,000 simulation steps were dedicated to system equilibration, followed by 100,000 production steps for data sampling and statistical analysis. During the production phase, trajectory files were continuously recorded to capture MD behaviors. Trajectories were analyzed in the Amorphous Cell module, and the system’s CED and δ were calculated. The solubility parameter δ, which characterizes intermolecular interactions in pure substances, was calculated according to Equation (1) [41]:

(1) $$\delta \;=\;\sqrt{\mathrm{CED}}\;=\;\sqrt{\frac{\Delta \mathrm{Ev}}{\mathrm{Vm}}}$$

CED represents the cohesive energy per unit volume, denoting the minimum energy required to physically separate molecules or atoms. Where ΔE_v_ is the enthalpy of vaporization, V_m_ is the molecular volume of the component.

### 4.3. Simulations of Solid–Liquid Lipid Carrier Mixture Systems

**Figure 10:**
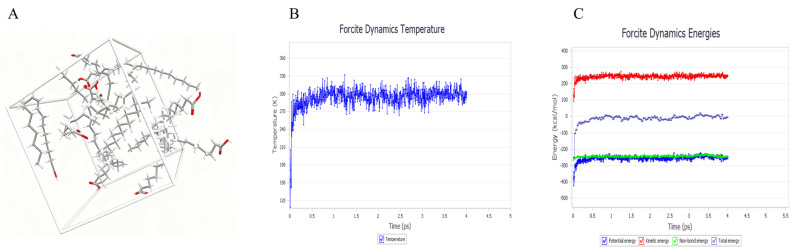
MD simulations results. (**A**) Three-dimensional model of the blend system; (**B**) Temperature versus time equilibrium curve; (**C**) Energy versus time equilibrium curve.

Factors influencing the drug-loading capacity of NLCs depend not only on the compatibility between the drug and the lipid carrier but also on the physicochemical structure of the blended lipid carrier, crystallization, and the phase transitions of solid lipids. Based on the solubility-parameter screening results, the subsequent step was to determine the ratio of solid to liquid lipids using mixing energy as the evaluation criterion [42]. The solid–liquid lipid ratio, as a core formulation variable [43], influences NLCs’ stability by regulating the phase behavior and crystal structure of the lipid matrix. During NLCs preparation, phase separation can occur among different lipids in the molten state, and their varying melting points influence the morphological forms and structural types of liquid and solid lipids within the cooled NLCs. The forms, structures, and interactions between liquid and solid lipids during NLC storage can affect solid lipid recrystallization and phase transitions. Therefore, the physicochemical structure, molar ratio, and compatibility between liquid and solid lipids determine the structural type of NLCs. The morphological structure of lipids within NLCs is closely related to their drug-loading capacity, release behavior, and stability. Such investigations are critical for achieving precise control over the structure of NLCs and further developing efficient, safe, and stable nanolipid-based drug delivery systems [44]. Using Flory-Huggins theory combined with MD simulations, we investigated how the compatibility of solid–liquid lipid blends affects the structural types and properties of NLCs. The structural units of the blend system underwent 5000 steps of molecular mechanics optimization until energy convergence. An NVT ensemble was selected, employing an Andersen thermostat at 298 K with a time step of 1.0 fs for MD simulations, totaling 15,000 simulation steps. Subsequently, annealing MD simulations were performed on the structural units optimized by the MD method. After each annealing cycle, the system was re-optimized by molecular mechanics. This process was repeated 10 times until the system energy stabilized. The configuration from the final optimization step served as the initial configuration for the simulation study, which yielded the three-dimensional model of the stearic acid-oleic acid blend structural unit (Figure 10A). The NVT ensemble was simulated at 298 K with a time step of 1 fs, totaling 180,000 simulation steps, and trajectory files were recorded every 100 steps. For the equilibrated blend system, trajectories were analyzed in the Amorphous Cell module to calculate the system’s CED and δ values. Two criteria were used to determine simulation equilibrium: (i) thermal equilibrium, where the standard deviation of temperature variation was less than 5% (Figure 10B); and (ii) energy equilibrium, characterized by small fluctuations around a constant value (Figure 10C). The target density of the blend structural unit was calculated according to Equation (2) [45] as follows:

(2) $$\rho_{blend}=\varphi_{A}\rho_{A}+(1-\varphi_{A})\rho_{B}$$

where $\varphi_{A}$ represents the volume fraction of lipid carrier *A* in the binary mixture system, $\rho_{A}$ and $\rho_{B}$ denote the true densities of lipid carriers *A* and *B*, respectively.

### 4.4. Formulation Optimization

Initial screening of relevant variables was carried out through single-factor experiments. The formulation parameters examined included drug-to-lipid ratio, solid–liquid lipid content, mass fraction of Poloxamer 188, homogenization pressure, and homogenization cycle count. Based on the results of these preliminary tests, a systematic optimization was performed using the BBD response surface methodology to explore the interactions among the key formulation factors. Three variables—drug-to-lipid ratio, solid–liquid lipid ratio, and Poloxamer 188 content—were selected for detailed investigation. A three-factor, three-level experimental design was constructed to evaluate the effects on EE% and particle size. This approach aimed to identify the optimal combination of formulation parameters to maximize EE% and minimize particle size for enhanced nanocarrier performance.

### 4.5. Preparation of SIN-NLCs

SIN-NLCs were prepared using high-pressure homogenization. A precise amount of SIN, together with the prescribed quantities of stearic acid (solid lipid) and oleic acid (liquid lipid), was placed in a clean beaker. An appropriate amount of anhydrous ethanol was added, and the mixture was heated in a constant-temperature water bath at 65–70 °C under magnetic stirring until a clear and transparent organic phase was obtained. Separately, 50 mL of Poloxamer 188 aqueous solution at a specific concentration was prepared and maintained in a 65–70 °C water bath, and stirred under the same conditions until a transparent and clear solution was obtained, serving as the aqueous phase. The organic phase was then added dropwise to the aqueous phase under magnetic stirring at the same temperature and maintained for 30 min. The initial emulsion was subsequently subjected to high-pressure homogenization. Cyclic homogenization was performed under high-pressure conditions to improve nanoparticle uniformity. The homogenized solution was cooled in a refrigerator, filtered through a 0.45 μm microporous membrane filter, and the filtrate was collected. Finally, purified water was added to adjust the suspension to a final volume of 50 mL, yielding the SIN-NLCs suspension.

### 4.6. Characterization of SIN-NLCs

#### 4.6.1. Particle Size and Zeta Potential

The particle size and zeta potential of SIN-NLCs were measured using a Malvern ZS90 DLS particle size analyzer. The instrument was preheated for 30 min, and measurement parameters were adjusted prior to analysis. Samples were equilibrated for 30 s at 25 °C before data acquisition. Each sample was measured in triplicate, and the average values were reported.

#### 4.6.2. EE%

Briefly, 1 mL of SIN-NLCs suspension was placed in an ultrafiltration tube (molecular weight cutoff: 10 kDa), centrifuged at 12,000 rpm (4 °C, rotor radius 6.4 cm) for 20 min, and the free drug content (W_free_) of SIN in the supernatant was quantified using a high-performance liquid chromatography (HPLC) system (2030, Shimadzu, Japan). The chromatographic conditions were as follows: Waters Symmetry C18 column (4.6 mm × 250 mm, 5 μm), mobile phase of methanol and 0.05 M sodium dihydrogen phosphate (20:80), flow rate of 1.0 mL/min, detection wavelength at 262 nm, column temperature maintained at 30 °C, and injection volume of 10 μL. Separately, 1 mL of SIN-NLCs suspension was accurately weighed, mixed with methanol, subjected to ultrasonic disruption, and the total drug content in the nanoparticles (W_total_) was determined using the same method. The encapsulation efficiency of SIN-NLCs was calculated according to Equation (3):

(3) $$\mathrm{EE}\%\;=\;(1-\frac{W_{\mathrm{free}}}{W_{\mathrm{total}}})\;\times \;100\%$$

Among these, W_total_ represents the total drug content in the NLCs, while W_free_ denotes the unencapsulated drug content in the ultrafiltrate.

#### 4.6.3. TEM

The microstructure of nanoparticles was observed using a transmission electron microscope (JEM-2100, JEOL, Tokyo, Japan). TEM was employed to observe the microstructure of the nanoparticles. A 10 μL sample was deposited onto a copper grid. The sample was stained with 10 μL of 2% phosphotungstic acid solution for 10 min. Excess staining solution was removed, and the sample was allowed to air dry. The TEM was operated at an accelerating voltage of 100 kV.

### 4.7. Spectral Analysis of SIN-NLCs

#### 4.7.1. XRD

X-ray diffraction (D8 Advance, Bruker AXS, Karlsruhe, Germany) was employed to investigate the crystallinity, crystal structure, and structural disorder of NLCs. The crystallinity, crystal structure, and structural disorder of NLCs were analyzed by examining their diffraction peaks. The crystal structure characteristics of the lipid carriers were determined from their XRD patterns. Samples including SIN, SIN-NLCs, physical mixtures (prepared at the same excipient ratio as the SIN-NLCs formulation), and solid–liquid mixed excipients were placed in sample holders for XRD scanning. The test conditions were as follows: room temperature; Cu target (Kα radiation, λ = 0.154187 nm); scanning voltage, 40 kV; tube current, 40 mA; scanning speed, 2°/min; sampling interval, 0.02°; and scanning range, 5–80°. No significant changes were observed in the XRD patterns beyond 30°.

#### 4.7.2. DSC

DSC was performed using a DSC instrument (Mettler DSC3). Samples included SIN, SIN-NLCs, physical mixtures, and solid–liquid mixed excipients. Approximately the appropriate amount of each sample was weighed, sealed in aluminum crucibles, and heated at a rate of 10 °C/min over the temperature range of 25–250 °C. The measurements were carried out under a nitrogen atmosphere. An empty crucible was used as a reference.

#### 4.7.3. FT-IR

FT-IR spectra were recorded on a Nicolet iS50 spectrometer (Thermo Scientific, Waltham, MA, USA). FT-IR is a technique for characterizing the molecular structure of substances. By analyzing the position and intensity of characteristic absorption peaks, the composition of lipid carriers can be determined, and changes in these peaks can be used to detect the crystalline form of drugs. SIN, SIN-NLCs, physical mixtures, solid–liquid mixed excipients, and KBr were placed in a mortar at a 1:100 ratio, ground uniformly, and then compressed into pellets. Samples were prepared using the KBr pellet method. Compression was performed at 5 tons of pressure for 2 min to form transparent pellets. The scanning range was 4000–400 cm^−1^, with a resolution of 4–8 cm^−1^.

### 4.8. In Vivo Pharmacodynamic Studies

#### 4.8.1. Ethical Statement

Thirty SPF grade healthy male SD rats weighing 200 ± 20 g were purchased from Liaoning Changsheng Biotechnology Co., Ltd. All animals were housed at the Animal Experiment Center of Changchun University of Chinese Medicine under controlled conditions at 25 ± 2 °C, 50 ± 5% relative humidity, and a 12 h light/dark cycle. Animals were acclimatized for one week before the experiments. All animal experiments in this study were approved by the Ethics Committee of Changchun University of Chinese Medicine.

#### 4.8.2. Complete Freund’s Adjuvant-Induced (CFA) and Animal Treatment

In rat models of RA, the central mechanism of complete Freund’s adjuvant–induced arthritis is to elicit an autoimmune inflammatory response that closely mimics the pathological characteristics of human RA. The Bacille Calmette–Guerin (BCG) vaccine was dissolved in sterile saline to prepare a 10 mg/mL solution. This solution was mixed with complete Freund’s adjuvant and emulsified thoroughly until a stable oil-in-water emulsion was formed. The stability of the emulsion was confirmed by dropping it into water; an intact droplet without dispersion indicated successful emulsification. Each rat was injected with 0.1 mL of the 10 mg/mL BCG-CFA emulsion into the left plantar region [46]. A 4.5 mL emulsion was prepared by dissolving 15 mg of BCG vaccine in 3 mL sterile saline and mixing with 1.5 mL of complete Freund’s adjuvant. The second immunization was performed on day 14. Rats were randomly divided into five groups (n = 6 per group): normal group, model group, DXM group, SIN group, and SIN-NLCs group. Treatment commenced on day 15, with daily oral gavage administration for 21 consecutive days in each group. Under sterile conditions, rats in the model group received subcutaneous injections of complete Freund’s adjuvant into the lateral region of the right thigh and the right hind paw pad. Concurrently, rats in the normal group received equivalent volumes of saline at the same sites. The doses were calculated based on body surface area normalization, using 216 mg/day of SIN as the human equivalent dose, which corresponds to approximately 19.44 mg/kg/day in rats [47]. Gastric gavage was performed once daily by administering 10 mL·kg^−1^ of the respective drug solution for 21 consecutive days. Both the normal group and the model group were administered an equivalent volume of distilled water by oral gavage. On the final day, blood was collected from the abdominal aorta of anesthetized rats. The ankle joints, spleen, and thymus tissues were immediately frozen in liquid nitrogen and then stored at −80 °C for subsequent analysis. Additionally, the thymus and spleen were excised from the euthanized rats and weighed immediately.

#### 4.8.3. Paw Edema Index, Arthritis Score, Immune Organ Indicators, Inflammatory Cytokine Detection, and HE Analysis in Rats

Spleen and thymus indices were used to assess immunomodulatory activity. The thymus and spleen indices were calculated according to the formula: Organ index = Organ weight (mg)/Body weight (g). IL-6, IL-1β, and TNF-α were measured using commercially available ELISA kits. Rat metatarsophalangeal joint tissues were immediately fixed in 4% paraformaldehyde, embedded in paraffin, sectioned at 5 μm, stained with HE, and examined histopathologically under a light microscope.

### 4.9. SPIP

The SPIP method was employed to investigate the absorption characteristics of SIN and SIN-NLCs in different intestinal segments of rats. For sample preparation, SIN-NLCs and SIN solution were diluted to 150 µg/mL in Krebs-Ringer (K-R) buffer [48]. Rats were anesthetized by intraperitoneal injection of 20% urethane solution and secured on a dissection table. Following loss of the pain reflex, a 3–4 cm midline abdominal incision was made. The intestinal contents were flushed with pre-warmed (37 °C) physiological saline. A cannula was inserted into the intestinal segment and secured with surgical thread. Donor and recipient vials were connected to each end of the intestinal segment via a peristaltic pump. The wound was covered with saline-soaked gauze to maintain moisture and kept warm under an infrared lamp throughout the procedure. The intestine was first perfused with blank buffer solution at 0.2 mL/min for 30 min to achieve steady-state conditions [49]. Subsequently, the intestine was perfused with SIN-NLCs or SIN solution at 0.2 mL/min, and perfusate samples were collected at 45, 60, 75, 90, 105, and 120 min. Collected perfusate was accurately measured, mixed with methanol, centrifuged at 12,000 rpm for 10 min, and the supernatant was filtered through a membrane. Aliquots (10 µL) were analyzed by HPLC. At the end of the experiment, rats were euthanized, and the intestinal segments between the cannulas were excised. The length and inner diameter of each segment were measured and recorded. The Ka and Papp were calculated using Equations (4) and (5) [50].

(4) $$K_{a}=(1-\frac{C_{out}}{C_{in}}\times \frac{V_{out}}{V_{in}})\times \frac{Q_{in}}{\pi r^{2}l}$$

(5) $$P_{app}=\frac{-Q_{in}\ln\left(\frac{C_{out}}{C_{in}}\times \frac{V_{out}}{V_{in}}\right)}{2\pi rl}$$

In the formula, *C_out_* and *C_in_* represent the drug concentrations at the outlet and inlet, respectively; *V_out_* and *V_in_* denote the perfusion fluid volumes in the receptor and donor vials, respectively; *Q* is the flow rate (mL/min), *l* is the intestinal length (cm), and *r* is the intestinal radius (cm).

## 5. Conclusions

This study innovatively integrated MD simulation with conventional formulation technology, markedly shortening the research and development cycle while reducing material and time costs, thus effectively overcoming the limitations of traditional trial-and-error screening methods. By combining molecular modeling with experimental validation in a bidirectional optimization strategy, the optimal formulation and preparation process of SIN-NLCs were successfully established. The resulting nanoparticles exhibited nanoscale dimensions, uniform dispersion, and high encapsulation efficiency. Experimental data demonstrated that SIN-NLCs significantly alleviated arthritic symptoms in rats and showed superior safety and biocompatibility compared with dexamethasone. Furthermore, SIN-NLCs improved intestinal absorption and permeability, underscoring the advantages of oral administration. Overall, nanostructured lipid carriers show substantial potential as oral nanodelivery systems. This work not only provides a feasible and efficient approach for optimizing SIN formulations but also offers a valuable reference for the formulation of other poorly soluble drugs. More importantly, the MD-simulation-guided carrier-design strategy established herein lays an essential methodological foundation for advancing pharmaceutical nanotechnology toward precision-oriented research and supports the future development of efficient, low-toxicity oral therapeutics for RA.

## Figures and Tables

**Table 1:** CED and δ values of SIN and carrier excipients.

Systems	CED ×10^8^(J/m^3^)	δ (Calculated Value) (J/cm^3^)^1/2^	δ (Literature Value) (J/cm^3^)^1/2^	Δδ (J/cm^3^)^1/2^
SIN	4.41	21.17	-	-
GMS	4.42	20.00	-	1.17
Stearic acid	4.28	20.52	-	0.65
Glycerol tristearate	3.27	18.44	17.37	3.33
α-tocopherol	3.75	17.52	-	3.65
Oleci acid	3.91	21.41	18.50	0.24
Glycerol tricaprylate	3.19	18.80	17.76	2.37
Poloxamer 188	4.91	19.20	-	1.97
Lecithin	5.19	23.21	-	2.04
PVA 0486	5.25	24.36	24.34	3.19
Ethanol	3.94	27.90	26.42	6.73
Water	2.46	48.26	47.04	27.09

**Table 2:** Response surface experiment factors and levels design table.

	Factors	Drug-To-Lipid Ratio (g/g)	Solid Lipid–Liquid Lipid (g/g)	Poloxamer 188 Content (%)
Levels				
−1		1:30	5:5	0.75
0		1:40	6:4	1.00
1		1:50	7:3	1.25

## Data Availability

Data are contained within the article.
